# Effects of mind-body exercises on depressive symptoms in older adults: a comprehensive umbrella review of the evidence

**DOI:** 10.3389/fpsyg.2026.1806563

**Published:** 2026-05-07

**Authors:** Fangbo Li, Qingfang Li, Jiafu Huang, Xuecheng Li, Zuosheng Lu

**Affiliations:** 1 School of Physical Education and Sports Science, South China Normal University, Guangzhou, China; 2 School of Sports Media, Guangzhou Sport University, Guangzhou, China; 3 Adapted Physical Activity + Laboratory, South China Normal University, Guangzhou, China

**Keywords:** depression, mind-body exercises, older adults, Qigong, Tai Chi, umbrella meta-analysis, yoga

## Abstract

**Background:**

Depression is common in older adults, often with suboptimal pharmacological responses. Mind-body exercises (MBEs) like Tai Chi, Yoga, and Qigong have emerged as potentially beneficial non-pharmacological interventions. However, existing systematic reviews report heterogeneous findings, necessitating a comprehensive synthesis of the available evidence.

**Methods:**

We systematically searched PubMed, Cochrane Library, Web of Science, and ScienceDirect to August 2025, following PRISMA and PRIOR guidelines. Systematic reviews and meta-analyses examining MBEs' effects on depressive symptoms in adults ≥60 years were included. The Corrected Covered Area (CCA) assessed overlap between reviews. Effect sizes were synthesized using random-effects modeling. Quality was assessed via AMSTAR-2 and GRADE.

**Results:**

Twenty-five reviews (>20,000 participants) were included, with moderate overlap (CCA = 6.98%). The synthesized evidence suggested moderate reductions in depressive symptoms (pooled SMD = −0.51, 95% CI: −0.66 to −0.35, *p* < 0.0001). Subgroup analyses showed larger effect sizes for Qigong (SMD = −0.49) vs. Yoga (SMD = −0.45) and Tai Chi (SMD = −0.34). However, substantial heterogeneity was observed (*I*^2^ = 90.89%). Sensitivity analyses confirmed robustness. Trim-and-fill adjustment suggested potential publication bias (adjusted SMD = −0.40).

**Conclusion:**

This umbrella meta-analysis provides moderate-certainty evidence that MBEs, particularly Qigong, may be associated with reductions in depressive symptoms in older adults, though the substantial heterogeneity (*I*^2^ = 90.89%) across included reviews indicates that pooled estimates should be interpreted as reflecting a general directional trend rather than definitive effect sizes. A quality-stratified sensitivity analysis restricted to High Confidence reviews yielded a stronger and homogeneous effect (SMD = −0.62, *I*^2^ = 0.0%). However, the predominance of lower-quality reviews and evidence of publication bias necessitate cautious interpretation. These findings provide a directional evidence synthesis to inform future high-quality research rather than definitive clinical recommendations.

**Systematic Trial Registration:**

https://www.crd.york.ac.uk/PROSPERO/view/CRD420251131812, Identifer [PROSPERO 2025 CRD420251131812].

## 1 Introduction

Depression and anxiety are among the most prevalent mental health disorders affecting aging populations worldwide, with profound implications for individuals' quality of life, physical health, and overall well-being (Hu et al., 2022; Ribeiro et al., 2020). Global estimates indicate that older adults are especially vulnerable to these disorders, often due to factors such as social isolation (Teo et al., 2023), chronic illnesses, cognitive decline (Guarnera et al., 2023), and life transitions (e.g., retirement or bereavement) (Yon et al., 2017). Notably, even mild symptoms of depression and anxiety in this population can impair daily functioning and increase the risk of more severe mental health disorders if left unaddressed (Cheng et al., 2024). Consequently, addressing these challenges is a critical public health priority.

Pharmacological treatments, including antidepressants and anxiolytics, are commonly prescribed for the management of depression and anxiety (De Luca et al., 2025; Crocco et al., 2017). However, these treatments are frequently associated with side effects, drug interactions, and limited long-term efficacy in older adults (Dickinson et al., 2010). Additionally, concerns regarding over-reliance on medication have prompted researchers and clinicians to explore non-pharmacological interventions as alternative or complementary approaches (Aguiñaga et al., 2018; Geiger et al., 2016). These interventions, including psychosocial therapies, physical activity programs, mindfulness-based practices, and lifestyle modifications, aim to offer holistic and sustainable solutions for mental health management in aging populations (Hall et al., 2016; Geiger et al., 2016).

Mind-body exercises (MBE), which encompass various movements and postures, such as musculoskeletal stretching, relaxation, breath control, and mental focus, have gained significant global popularity (Takemura et al., 2020). Several studies have investigated the effects of MBE (e.g., yoga, qigong, and tai chi) on people with various conditions and disabilities, concluding that these practices can improve quality of life, mood, stress levels, immune function, and social functioning (Zeng et al., 2014; Buffart et al., 2012; Huang et al., 2023, 2021). Compared to other forms of exercise, MBE integrate physical exercise with breathing and deep relaxation techniques, thereby enhancing physical function along with emotional and psychological awareness, potentially offering greater benefits than other exercise forms (Li Y. et al., 2025). In particular, MBE are gentle, require minimal equipment or external assistance, and are easy to perform and learn, irrespective of location or time (Zhang et al., 2023). As a result, they have shown high adherence among older adults, who frequently suffer from chronic illnesses or limited mobility (Li G. et al., 2025). Studies have demonstrated that MBE has achieved a high completion rate in the older adults with chronic diseases (e.g., chronic obstructive pulmonary disease and heart failure), and significantly reduces anxiety and depression (Xiao and Zhuang, 2015; Li et al., 2020; Cheung et al., 2021).

In recent years, several randomized controlled trials (RCTs) and meta-analyses have been published examining the effects of MBE on depression in older adults, although the conclusions remain inconsistent. Both Li and Zhang's studies concluded that MBE had negligible effects on improving depressive symptoms in the older adults, while Wang et al.'s study found that MBE had significant effects (Li et al., 2022; Zhang et al., 2019; Wang et al., 2022). This indicates the need for further research to explore the actual effects of MBE and reach a definitive conclusion. Umbrella review, also known as a system of systematic reviews, is a research method that systematically reassesses all existing systematic reviews and meta-analyses on a specific medical research topic, thereby yielding more reliable conclusions. The umbrella review synthesizes evidence from systematic reviews into a comprehensive, accessible report (Ioannidis, 2009; Papatheodorou, 2019). Two prior umbrella reviews of the effects of exercise on depression in older adults have yielded consistent results, demonstrating that exercise is both safe and effective in alleviating depressive symptoms in this population (Catalan-Matamoros et al., 2016; Bigarella et al., 2022). However, to the best of our knowledge, no direct quantitative comparison has been made regarding the effects of MBE on depression in older adults. Furthermore, the quality of the meta-analyses and included randomized controlled trials has yet to be assessed, which is an essential step before making confident treatment recommendations (Köhler-Forsberg et al., 2023). Therefore, this umbrella review aims to evaluate the effects of MBE (I) on depressive symptoms (O) in older adults aged ≥60 years, compared to usual care or a control group (P), to inform future research in this field.

## 2 Methods

### 2.1 Protocol and registration

This review was reported in accordance with Preferred Reporting Items for Systematic Reviews and Meta-Analyses (PRISMA)32 and Preferred Reporting Items for Overviews of Reviews (PRIOR)33 guidelines (Shamseer et al., 2015; Gates et al., 2022). The protocol was preregistered on PROSPERO (ID: CRD420251131812).

### 2.2 Search strategy

A comprehensive literature search was performed across PubMed, Cochrane Library, Web of Science and Science Direct from inception to August 23, 2025. Search terms were grouped into three categories: (1) “mind-body exercise” OR “Tai Chi” OR “Yoga” OR “Qigong”; (2) “depression” OR “depressive symptoms”; and (3) “older adults” OR “elderly” OR “geriatric” AND (“meta-analysis” OR “systematic review”). The asterisk (^*^) was used for truncation to enhance sensitivity. Additionally, reference lists of included studies were manually screened for further eligible reviews. (e.g., PubMed: (“mind-body exercise^*^” [tiab] OR “Tai Chi” [tiab] OR “Yoga” [tiab] OR “Qigong” [tiab]) AND (“depression^*^” [tiab] OR “depressive symptom^*^” [tiab] OR “depression” [mh]) AND (“older adult^*^” [tiab] OR “elderly” [tiab] OR “geriatric^*^” [tiab] OR “aged” [mh]) AND (“meta-analysis” [pt] OR “systematic review^*^” [tiab])).

### 2.3 Inclusion and exclusion criteria

The objective was to evaluate the effects of MBE (I) on depressive symptoms (O) in older adults ≥60 years (P) compared to usual care or controls (C). Studies were included if they met the following criteria: (1) study type was a systematic review or meta-analysis (including network meta reviews); (2) participants were older adults aged 60 years and above, or included other age groups with a separate older adult subgroup analysis or ≥75% older adult representation (Singh et al., 2025); (3) interventions involved at least one type of MBE (e.g., Yoga, Tai Chi, and/or Qigong); (4) outcomes included improvements in depression symptoms, measured by standardized scales (e.g., Geriatric Depression Scale (GDS), Beck Depression Inventory (BDI), Hamilton Depression Rating Scale (HAM-D), Geriatric Anxiety Inventory (GAI), State-Trait Anxiety Inventory (STAI)); and (5) English and Chinese peer-reviewed journal articles were included. Studies were excluded if: (1) they were not systematic reviews or meta-analyses (e.g., research articles, case reports); (2) they did not focus on older adults or lacked separable older adult data; (3) they failed to report depression outcomes; or (4) they were not published in English or Chinese.

### 2.4 Study selection

After retrieving records from the databases, search results were imported into EndNote and duplicates were removed. Titles and abstracts were screened against inclusion criteria by two independent reviewers. Full texts of potentially eligible studies were then assessed for inclusion, with discrepancies resolved through consensus or consultation with a third reviewer.

### 2.5 Data extraction process

Data were extracted independently by two reviewers using a standardized form, including first author, publication year, sample size, participant age range, intervention details, SMD, 95% CI, and heterogeneity metrics. Disagreements were adjudicated by a third reviewer. Missing data assumed not available if not reported.

### 2.6 Risk of bias assessment

The methodological quality was assessed using the AMSTAR-2 tool (Shea et al., 2017), evaluating domains such as protocol registration and bias management. It comprises 16 items, addressing aspects such as the clarity of the research question, reporting of the outcome measures of interest, the comprehensiveness of the literature search, database breadth, clarity of the study selection criteria, independence in data extraction, independence in study quality assessment, the appropriateness of the data synthesis method (considering statistical and clinical heterogeneity), thorough reporting of study results, study screening, verification of data extraction and quality assessment, evaluation of reporting bias, interpretation of study results, alignment with objectives, funding source declaration, disclosure of conflicts of interest, and plans for future revisions or updates. The certainty of evidence was graded using the Grading of Recommendations Assessment, Development, and Evaluation (GRADE) (Guyatt et al., 2011) approach, considering factors such as risk of bias, inconsistency, and publication bias. Two reviewers (FB and QF) independently evaluated the study quality of included studies. Discrepancies were resolved through discussion and consensus.

### 2.7 Statistical analyses

The Corrected covered area (CCA) method was used to assess the degree of overlap among the trials included in each systematic review and meta-analysis (Kirvalidze et al., 2023). A CCA score of 0% indicates that all included reviews consist entirely of independent trials, while a score of 100% indicates that all reviews share the same trials. The specific quantitative criteria for overlap are as follows: 0%-5% represents slight overlap, 6%−10% is considered moderate overlap, 11%−15% indicates high overlap, and >15% indicates very high overlap. If the overlap exceeds 15%, it is recommended to implement strategies to reduce the overlap before proceeding with the umbrella meta-analysis (Hennessy and Johnson, 2020).

Data were synthesized using a random-effects model with the DerSimonian-Laird estimator for tau^2^. Effect sizes were synthesized as standardized mean differences (SMDs) using a random-effects model. Where alternative metrics, such as weighted mean differences (WMDs), were reported, these were converted to SMDs by dividing the WMD by the pooled standard deviation, calculated from available data or estimated from confidence intervals (Higgins et al., 2019). Effect sizes interpreted according to Cohen's criteria (small: 0.2-0.5, medium: 0.5-0.8, large: >0.8). Heterogeneity was assessed using the Q test and *I*^2^ (low: < 25%, medium: 50%, high: >75%) (Deeks et al., 2019). Subgroup analyses were conducted based on intervention type (e.g., Yoga, Tai Chi), and sensitivity analyses included leave-one-out and outlier exclusion (SMD > |2|) (Deeks et al., 2019). Publication bias was assessed using Egger's test, trim-and-fill method, and funnel plots for visual inspection. Additionally, a quality-stratified sensitivity analysis was conducted by restricting the pooled analysis to reviews rated as High Confidence by AMSTAR-2, to assess the influence of methodological quality on pooled estimates. Analyses were performed using the “metafor” package in R (version 4.3).

## 3 Results

This umbrella meta-analysis synthesized data from 25 systematic reviews and meta-analyses, including a total of over 20,000 participants from diverse global settings, to evaluate the effects of MBE on depressive symptoms in older adults (aged ≥60 years). The study selection process, outlined in the PRISMA flow diagram, identified these reviews through a comprehensive search conducted until August 23, 2025.

### 3.1 Study selection

**Figure 1:**
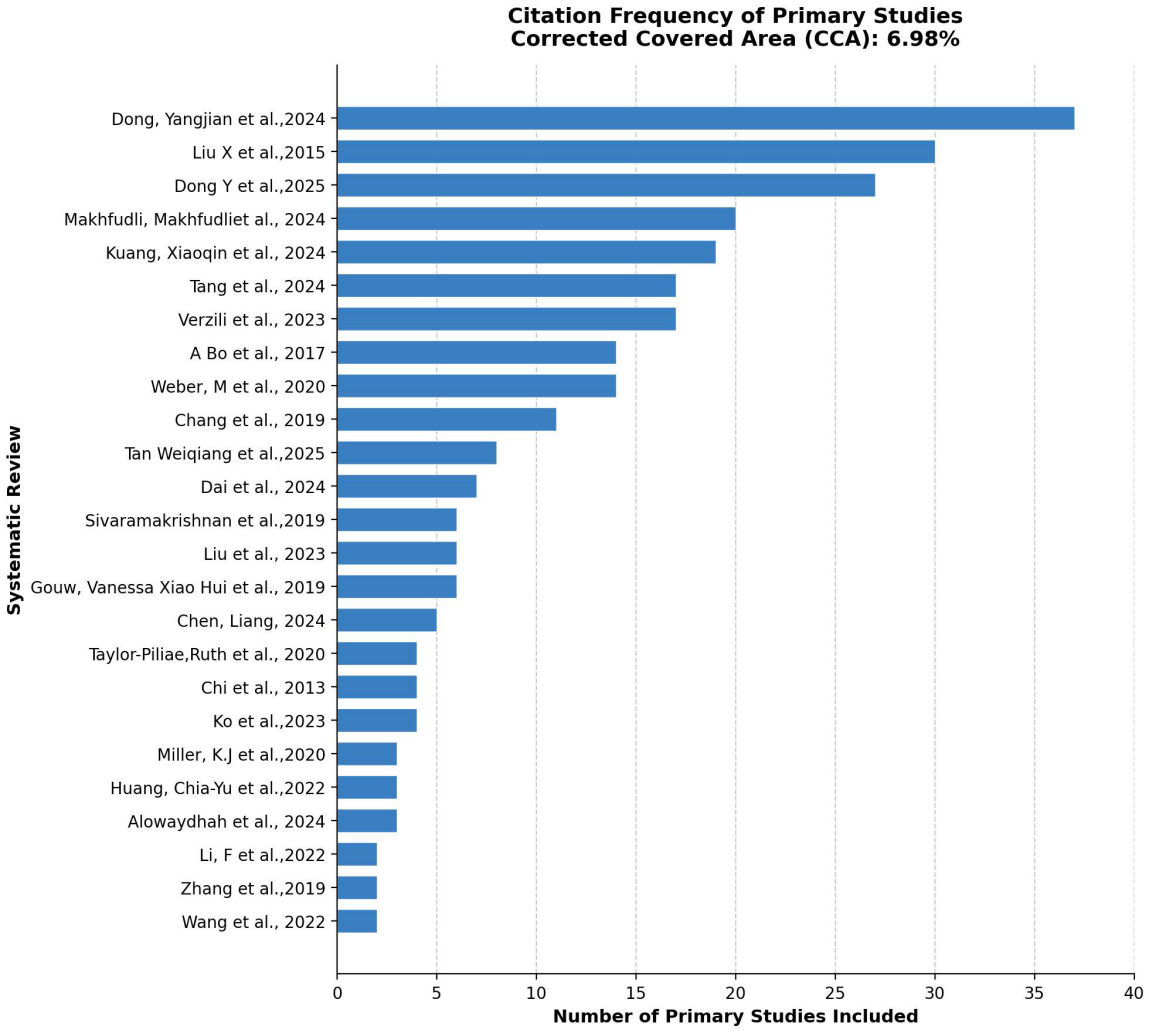
Citation frequency of primary studies across systematic reviews.

**Figure 2:**
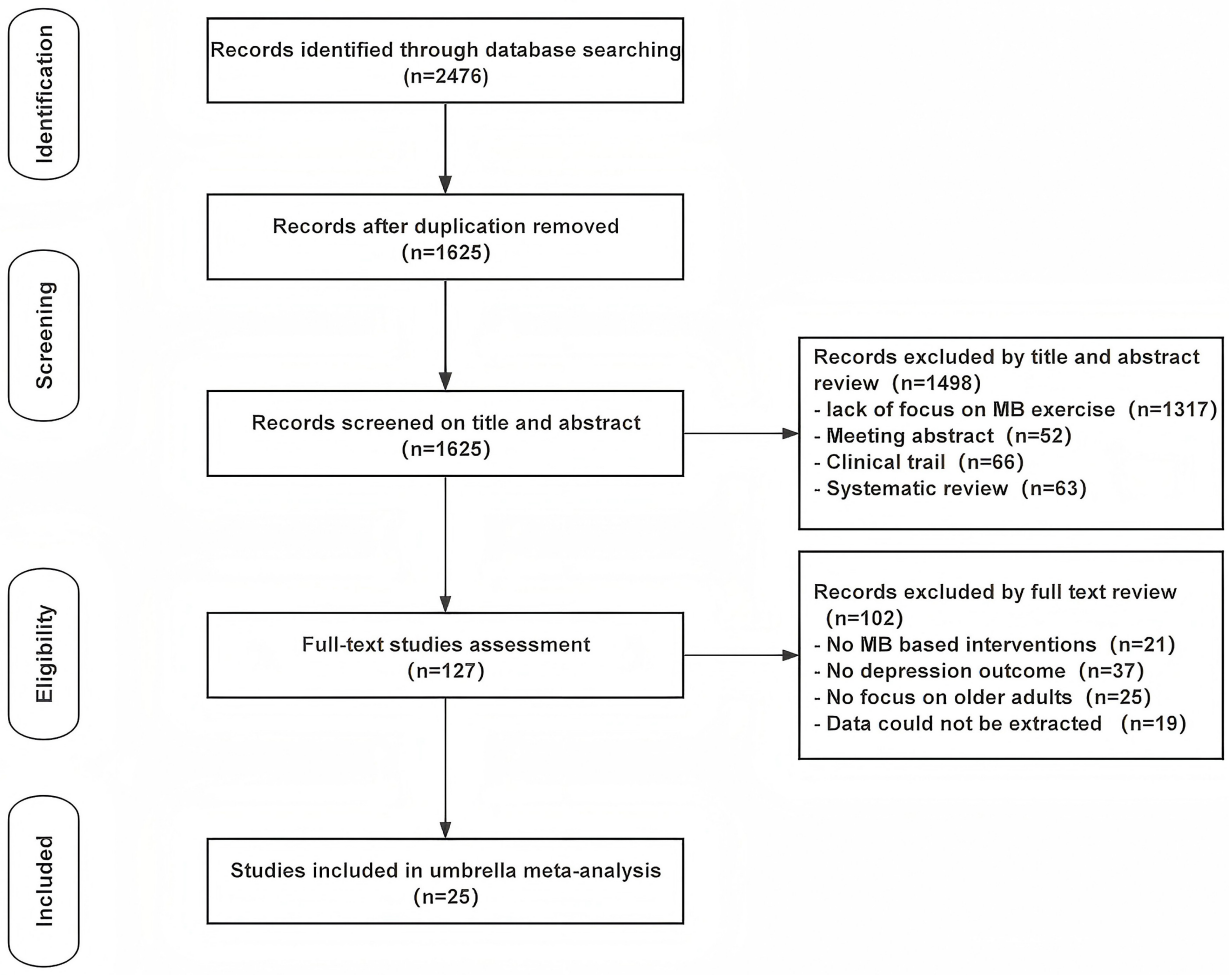
PRISMA flow diagram of study selection.

An initial literature search across the specified databases identified 2,476 publications. After excluding duplicate records, 1,625 unique articles advanced to the title and abstract screening phase. The subsequent evaluation resulted in 127 studies being selected for full-text assessment based on predefined inclusion criteria. Ultimately, 25 articles met the eligibility criteria, with a CCA of 6.98% (Figure 1), indicating moderate overlap among the primary studies included. Although this exceeds the threshold for slight overlap (< 6%), it remains below the 15% threshold above which overlap-reduction strategies are recommended prior to pooling (Hennessy and Johnson, 2020), thereby supporting the validity of the quantitative synthesis. The flow diagram is shown in Figure 2.

### 3.2 Study characteristics

The included reviews were published between 2013 and 2025. Table 1 summarizes key characteristics, including the first author, publication year, study design (all RCT-based meta-analyses), population (older adults aged >60 years, often with chronic diseases such as depression, mild cognitive impairment, or Parkinson's disease), interventions (Tai Chin = 9, Yogan = 5, Qigongn = 5, mixed MBEn = 10; some reviews examined multiple modalities, hence totals exceed 25), comparators (usual care, education, waiting list, health education, daily walking, standard care or active controls), primary outcomes (depressive symptoms assessed using GDS, BDI, HAM-D, CES-D), sample sizes (range: 116–3,228 participants), and the number of included studies (range: 2–38).

**Table 1:** Overview of all included systematic reviews and meta-analyses.

Author	Study design	Study population	Intervention		Sample size	Number of studies	Heterogeneity *I^2^*	AMSTAR-2
			Experimental	Control				
(Liu et al. 2015)	RCT	No restrictions on mean age (mean age greater than or equal to 60 years) or co-morbidities (defined as any additional medical condition other than depression)	Qigong or Tai Chi	Usual care control, other exercises, education, miscellaneous intervention	Qigong 1,425 Tai Chi 903	30	Qigong 84% Tai Chi 58%	Critically low confidence
(Tan et al. 2025)	RCT	Individuals with Parkinson's disease under clinical diagnosis	Tai Chi, Qigong, Baduanjin, Wuqinxi, and Yijinjing	Nonintervention, Conventional exercise, Daily walking	209 patients in the experimental groups and 201 patients in the control groups	8	92%	Low confidence
(Chi et al. 2013)	RCT	Older adults (aged 55 years or older) with certain depressive symptoms as measured by several rating scales	Tai Chi	No exercise, assignment to a waiting list, and health education	139 patients in the experimental groups and 114 patients in the control groups	4	0	Critically low confidence
(Verzili et al. 2023)	RCT	Elderly	Various types of Yoga	Waiting lists, or active controls	1,998 participants	17	77.40%	Critically low confidence
(Chang et al. 2019)	RCT	Older adults aged 62–83 years with depressive symptoms, frailty or chronic medical illnesses	Qigong exercise	Active control (e.g., daily walking, yoga, exercise therapy) or non-active	Total 1,282 (823 males, 459 females; from 14 RCTs)	14	Depression vs. active: 0%; vs. non-active: 86%	Critically low confidence
(Tang et al. 2024)	RCT	Older adults (mean age ≥60 years) with depressive symptoms	Qigong, Tai Chi, Yoga	Non-intervention, usual care, waitlist, health education, or head-to-head	2,895 participants	Qigong:5 Tai Chi:5 Yoga:7	Qigong 51% Tai Chi 29% Yoga 13%	Low confidence
(Dai et al. 2024)	RCT	Older adults (aged ≥60 years) with depressive episodes or symptoms	Multiple exercise types, including Qigong, Otago Exercise, Yoga, Tai Chi	Usual care, no treatment, low-intensity daily activities, waitlist, or non-interventional exercise	Total 3,238 participants	7	NA	High confidence
(Wang et al. 2022)	RCT	Participants with dementia (mean age = 82.0)	Tai Chi, aromatherapy, yoga, biofeedback	Standard care, placebo, and other types of interventions	116 participants	2	90.40%	High confidence
(Dong et al. 2025)	RCT	Old adults (60 years or older);	Tai Chi, Qigong, Wu Qin Xi, Ba Duan Jin, or Yi Jinjing	No exercise, daily activities, health education, medication, or low-intensity exercise;	1,179 patients in the experimental groups and 1,212 patients in the control groups	28	85%	High confidence
(Chen and Kim 2024)	RCT	Participants with mild cognitive impairment; average age of 71.83 years	Mind–body exercise interventions (e.g., Kundalini yoga and Chinese square dance)	Included no treatment, usual care, placebo, waitlist, or health education	251 patients in the experimental groups and 236 patients in the control groups	6	62%	Low confidence
(Makhfudli et al. 2024)	RCT	Older adults aged 60 years old or older living in the community or any type of long-term care facility	MBEs such as tai chi, yoga, qigong, and Pilates, either as standalone or combined practices	Usual care or non-exercise interventions	945 were assigned to the control group and 998 to the intervention group	20	73.50%	High confidence
(Ko et al. 2023)	RCT	The mean age of the participants ranged from 62 to 92 years.	Five types of yoga	Regular daily activities, wait-list, stretching exercise, education booklets	162 were assigned to the control group and 166 to the intervention group	4	42%	Critically low confidence
(Sivaramakrishnan et al. 2019)	RCT	Participants in the studies ranged from 61.0 years to 83.8 years	Eight types of yoga	Inactive controls	450 participants	6	57.09%	Low confidence
(Alowaydhah et al. 2024)	RCT	Individuals of either gender who were aged 65 or above.	Qigong and Tai Chi	Usual care, different types of interventions, or wait-list control	102 were assigned to the control group and 86 to the intervention group	3	82%	Critically low confidence
(Zhang et al. 2019)	RCT	Older adults (≥60 years) with MCI	Tai chi	Usual physical activity and usual care, or very low intensity of activity/exercise and other social activities	231 were assigned to the control group and 363 to the intervention group	2	0	Critically low confidence
(Bo et al. 2017)	RCT	Ages ranged from 60 and up	Mind–body interventions: Tai Chi, Qi Gong, yoga	No treatment standard care or non-therapeutic activities	1,019 participants	14	88%	Critically low confidence
(Weber et al. 2020)	RCT	Participants aged 59 years and above (72.2 ± 7.3)	Tai Chi, Qigong, Yoga or Pilates	No treatment/normal lifestyle/activities of daily living	3,224 participants	14	65%	Low confidence
(Dong et al. 2024)	RCT	Old adults aged ≥60 years, regardless of gender, duration of illness, or source of cases	Tai Chi, Qigong, Yoga, Dance, and Pilates	Routine daily activities, health education, physical exercise, and routine care	2,974 participants	38	80%	Low confidence
(Kuang et al. 2023)	RCT	Elderly individuals aged ≥60years	Various types of Tai Chi	Waiting list, usual care	765 were assigned to the control group and 815 to the intervention group	20	87%	High confidence
(Gouw et al. 2019)	RCT	Mean ages ranging from 63.9 ± 7.6 to 83.33 ± 6.30	Various forms of internal Qigong	No intervention, wait-list control, health education, routine activity, standard care, newspaper reading, and walking	340 were assigned to the control group and 218 to the intervention group	6	61%	Critically low confidence
(Huang et al. 2022)	RCT	The participants had a minimum age of ≥ 60 years and were diagnosed with frailty or sarcopenia	Tai Chi	No exercise or routine activity	58 in the treatment group and 64 in the control group	3	84%	High confidence
(Liu et al. 2023)	RCT	Older adults with MCI, all were older than 60 years of age	Mind-body exercise: Tai Chi, Qigong, Yoga, Dance	Usual care	129 in the treatment group and 125 in the control group	6	NA	Low confidence
(Miller et al. 2020)	RCT	Clinically depressed adults aged >65 years	Mind-body exercise: Tai Chi, Qigong	Wait-list control	Total of 596 participants (321 treatment and 275 controls)	3	NA	Low confidence
(Li et al. 2022)	RCT	Older adults (age: 60 years and older)	Tai Chi	Usual/standard care or standard education	*n =* 272	2	0	Low confidence
(Taylor-Piliae and Finley 2020)	RCT	Average 68 years old and most were men (72%)	Tai Chi	Usual care, health education-control	106 in the treatment group and 103 in the control group	4	0	High confidence

### 3.3 Methodological quality of included reviews

The methodological quality of the 25 included reviews was assessed using the AMSTAR-2 tool, revealing considerable variability in rigor (Table 2). Nine reviews were classified as critically low confidence, primarily due to deficiencies such as the absence of registered protocols, incomplete search strategies, and inadequate evaluation of risk of bias in the included studies (Liu et al., 2015; Chi et al., 2013; Verzili et al., 2023; Chang et al., 2019; Ko et al., 2023; Alowaydhah et al., 2024; Zhang et al., 2019; Bo et al., 2017; Gouw et al., 2019). Another 9 reviews were rated as low confidence, often due to a lack of clear justification for study exclusions, insufficient detail in data extraction procedures, or incomplete reporting of funding sources (Tan et al., 2025; Tang et al., 2024; Chen and Kim, 2024; Sivaramakrishnan et al., 2019; Weber et al., 2020; Dong et al., 2024; Liu et al., 2023; Miller et al., 2020; Li et al., 2022). In contrast, 7 reviews achieved high confidence, demonstrating adherence to most AMSTAR-2 criteria, including comprehensive investigations of heterogeneity and robust bias assessments (Dai et al., 2024; Wang et al., 2022; Dong et al., 2025; Makhfudli et al., 2024; Kuang et al., 2023; Huang et al., 2022; Taylor-Piliae and Finley, 2020). Common limitations across the reviews included unclear management of conflicts of interest and limited exploration of publication bias implications.

**Table 2:** AMSTAR-2 of all included systematic reviews and meta-analyses.

Author, year	1	2	3	4	5	6	7	8	9	10	11	12	13	14	15	16	Overall (AMSTAR-2)	Quality of evidence (GRADE)
(Liu et al. 2015)	Y	N	Y	Y	Y	PY	Y	Y	N	PY	Y	Y	Y	Y	Y	Y	Critically low confidence	Very low
(Tan et al. 2025)	Y	Y	N	Y	Y	Y	N	Y	Y	Y	Y	Y	Y	Y	Y	Y	Low confidence	Low
(Chi et al. 2013)	Y	Y	PY	Y	Y	Y	Y	Y	Y	N	Y	Y	Y	Y	N	PY	Critically low confidence	Low
(Verzili et al. 2023)	Y	N	N	Y	Y	Y	N	Y	Y	N	Y	Y	Y	Y	PY	Y	Critically low confidence	Moderate
(Chang et al. 2019)	Y	N	Y	PY	Y	Y	Y	Y	Y	N	Y	Y	Y	Y	N	Y	Critically low confidence	Moderate
(Tang et al. 2024)	Y	Y	Y	Y	Y	Y	Y	Y	Y	PY	Y	Y	Y	Y	Y	Y	Low confidence	Moderate
(Dai et al. 2024)	Y	Y	Y	Y	Y	Y	Y	Y	Y	N	Y	Y	Y	Y	Y	Y	High confidence	Moderate
(Wang et al. 2022)	Y	Y	Y	Y	Y	Y	Y	Y	Y	N	Y	Y	Y	Y	Y	Y	High confidence	Moderate
(Dong et al. 2025)	Y	Y	Y	Y	Y	Y	Y	Y	Y	N	Y	Y	Y	Y	Y	Y	High confidence	Moderate
(Chen and Kim 2024)	Y	Y	Y	Y	Y	Y	N	Y	Y	N	Y	Y	Y	Y	Y	Y	Low confidence	Low
(Makhfudli et al. 2024)	Y	Y	Y	Y	Y	Y	Y	Y	Y	N	Y	Y	Y	Y	Y	Y	High confidence	Moderate
(Ko et al. 2023)	Y	N	Y	Y	Y	Y	Y	Y	Y	N	Y	Y	Y	Y	N	Y	Critically low confidence	Low
(Sivaramakrishnan et al. 2019)	Y	Y	Y	Y	Y	Y	Y	Y	Y	N	Y	Y	Y	Y	N	Y	Low confidence	Low
(Alowaydhah et al. 2024)	Y	Y	Y	Y	Y	Y	N	Y	Y	N	Y	Y	Y	Y	N	Y	Critically low confidence	Low
(Zhang et al. 2019)	Y	N	Y	Y	Y	Y	Y	Y	Y	N	Y	Y	Y	Y	N	Y	Critically low confidence	Low
(Bo et al. 2017)	Y	N	Y	Y	Y	Y	N	Y	Y	N	Y	Y	Y	Y	Y	Y	Critically low confidence	Low
(Weber et al. 2020)	Y	N	Y	Y	Y	Y	PY	Y	Y	N	Y	Y	Y	Y	Y	Y	Low confidence	Low
(Dong et al. 2024)	Y	Y	Y	Y	Y	Y	N	Y	Y	N	Y	Y	Y	Y	Y	Y	Low confidence	Low
(Kuang et al. 2023)	Y	Y	Y	Y	Y	Y	PY	Y	Y	N	Y	PY	Y	Y	Y	Y	High confidence	Moderate
(Gouw et al. 2019)	Y	N	Y	Y	Y	Y	N	Y	Y	N	Y	PY	Y	Y	N	Y	Critically low confidence	Very low
(Huang et al. 2022)	Y	Y	Y	Y	Y	Y	Y	Y	Y	N	Y	Y	Y	Y	Y	Y	High confidence	Moderate
(Liu et al. 2023)	Y	Y	Y	Y	Y	Y	N	Y	Y	N	Y	Y	Y	Y	Y	Y	Low confidence	Low
(Miller et al. 2020)	Y	Y	Y	Y	Y	Y	N	Y	Y	N	Y	Y	Y	Y	Y	Y	Low confidence	Low
(Li et al. 2022)	Y	PY	Y	Y	Y	Y	Y	Y	Y	N	Y	Y	Y	Y	N	Y	Low confidence	Low
(Taylor-Piliae and Finley 2020)	Y	N	Y	Y	Y	Y	Y	Y	Y	N	Y	Y	Y	Y	N	N	High confidence	Moderate

AMSTAR (A Measurement Tool to Assess systematic Reviews) GRADE (Grading of Recommendations Assessment, Development and Evaluation).

The GRADE framework was used to assess the certainty of evidence, incorporating AMSTAR-2 ratings and the latest evaluations (Table 2). Overall, the certainty of evidence for the umbrella synthesis was rated as moderate. This assessment started with a high baseline, reflecting the inclusion of meta-analyses of randomized controlled trials (RCTs), but was downgraded by one level due to significant inconsistency (*I*^2^ = 90.89% across the pooled analysis) and another level due to evidence of publication bias (Egger's test, *p* < 0.0001). No further downgrades were required for indirectness, given the direct applicability to older adult populations, or for imprecision, supported by adequate sample sizes and confidence interval widths.

Individual review ratings ranged from very low—exemplified by Liu and Gouw (Gouw et al., 2019; Li et al., 2022), where critical methodological flaws and bias undermined reliability—to moderate, as seen in Verzili, Chang, and Dai (Chang et al., 2019; Dai et al., 2024; Verzili et al., 2023), where quality was affected by heterogeneity or bias concerns. Subgroup analyses further highlighted this variability: evidence for Tai Chi, as seen in (Taylor-Piliae and Finley 2020), achieved a moderate rating with low heterogeneity (*I*^2^ = 0%), while mixed interventions, as seen in (Tan et al. 2025), were rated lower due to high heterogeneity (*I*^2^ = 92%). These findings emphasize the influence of methodological quality on evidence certainty and underscore the need for cautious interpretation, particularly for reviews with critically low confidence ratings.

### 3.4 Overall effect

**Figure 3:**
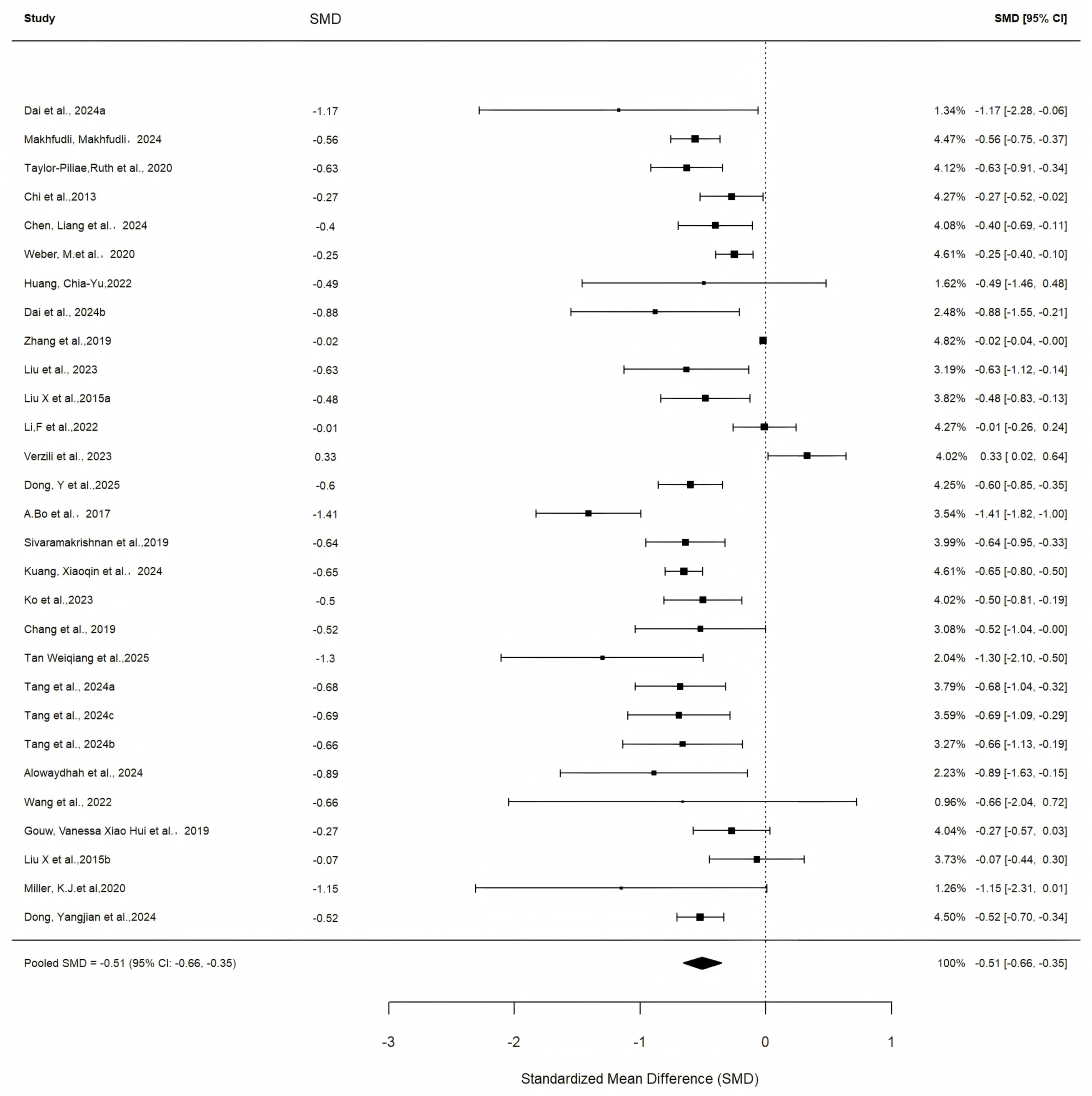
Forest plot of the overall effect of mind-body exercises on depressive symptoms in older adults.

The random-effects model yielded a pooled standardized mean difference (SMD) of −0.51 (95% CI: −0.66 to −0.35, z = −6.5436, *p* < 0.0001), suggesting a moderate reduction in depressive symptoms, though the substantial heterogeneity (*I*^2^ = 90.89%) necessitates cautious interpretation of this estimate, as it may reflect variability in study quality and intervention protocols rather than uniform clinical effects (Figure 3). Significant heterogeneity was observed (Q = 307.3163, df = 29, *p* < 0.0001), with *I*^2^ = 90.89%, H^2^ = 10.98, tau^2^ = 0.124 (SE = 0.0933), and tau = 0.3516, indicating substantial variability potentially attributable to intervention differences or population heterogeneity.

### 3.5 Subgroup analysis

**Figure 4:**
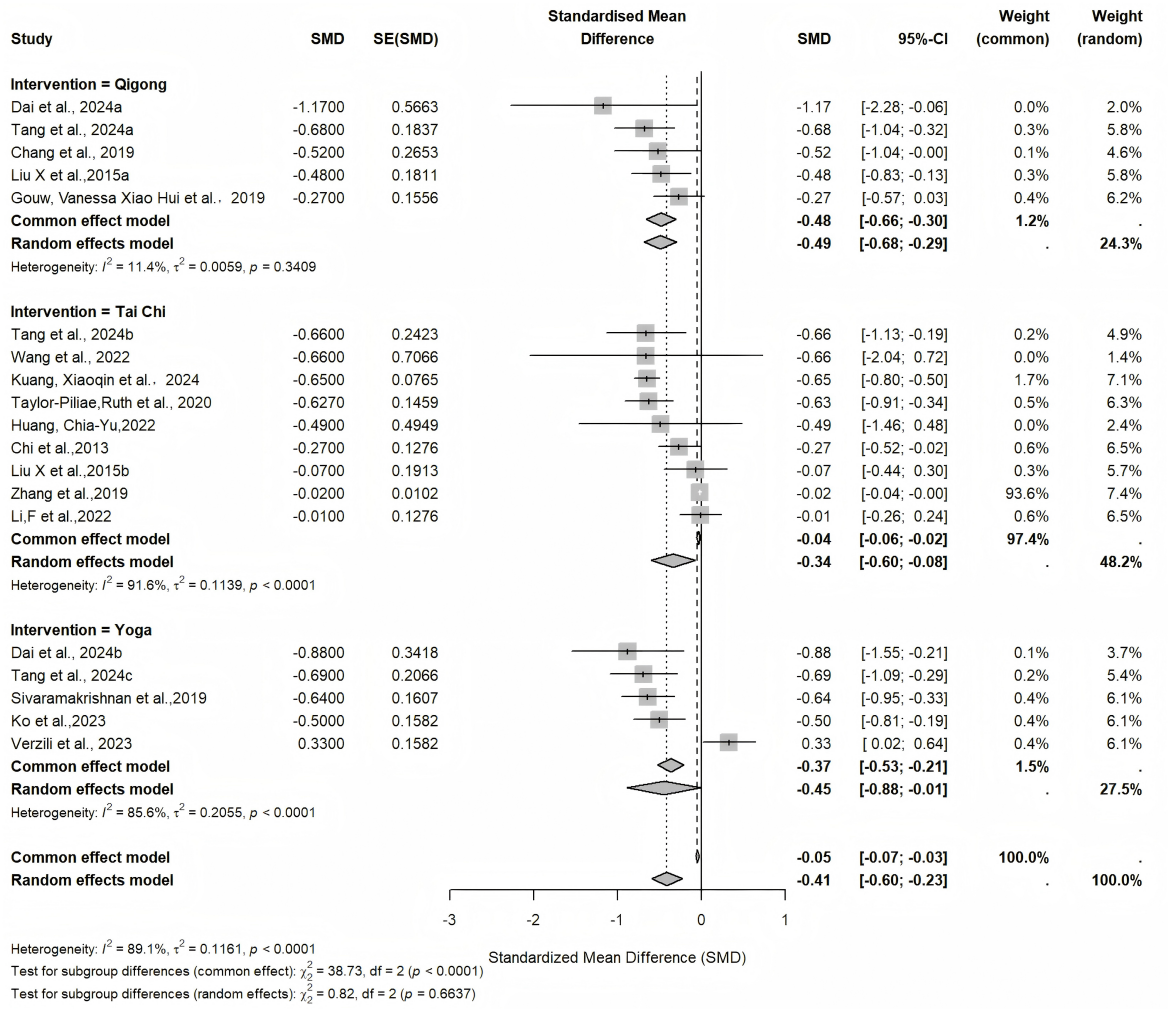
Forest plot of subgroup analyses by intervention type (Qigong, Tai Chi, and Yoga).

Subgroup analyses by intervention type revealed varying effects: Tai Chi (n = 9) yielded an SMD of −0.34 (95% CI: −0.60 to −0.08, *p* < 0.05, *I*^2^ = 91.6%); Yoga (n = 5) produced an SMD of −0.45 (95% CI: −0.88 to −0.01, *p* < 0.05, *I*^2^ = 85.6%); Qigong (n = 5) resulted in an SMD of −0.49 (95% CI: −0.68 to −0.29, *p* < 0.001, *I*^2^ = 11.4%). These findings suggest that Qigong may provide the strongest benefits, although heterogeneity remained high across subgroups (Figure 4).

### 3.6 Publication bias

**Figure 5:**
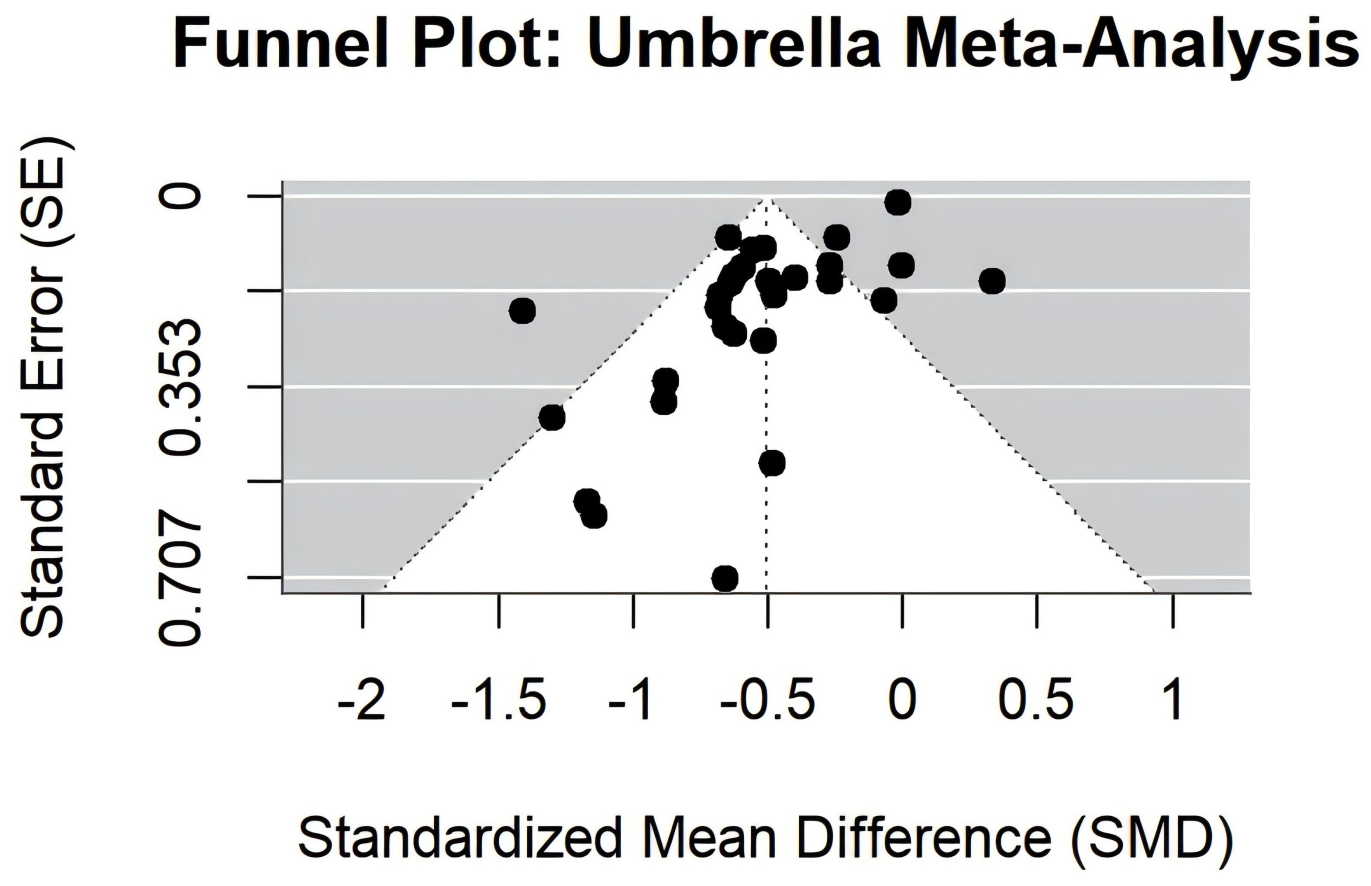
Funnel plot for publication bias assessment.

**Figure 6:**
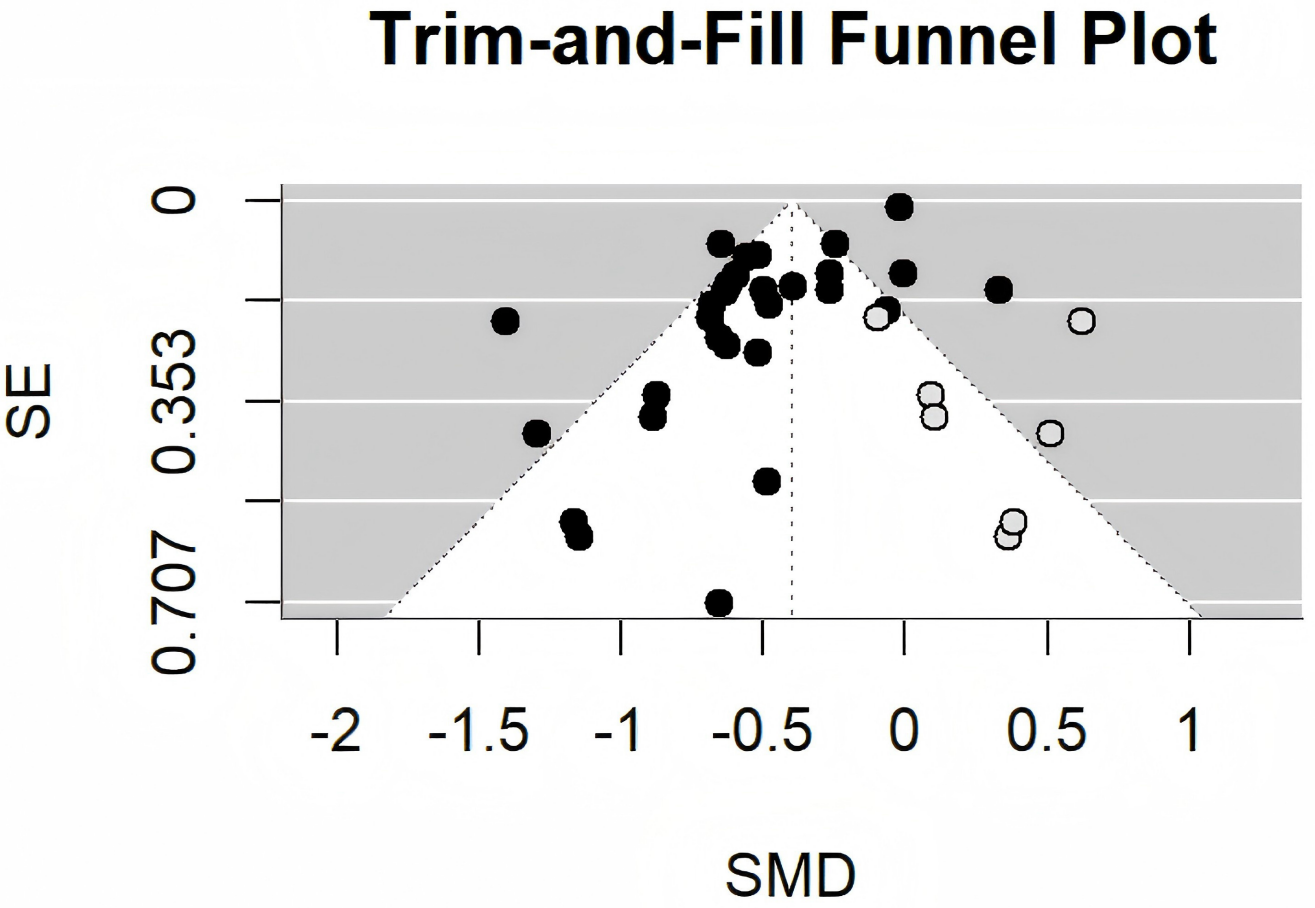
Trim-and-fill funnel plot for publication bias adjustment.

Egger's test revealed significant funnel plot asymmetry (t = −6.5073, df = 27, *p* < 0.0001), with a limit estimate intercept of −0.0003 (95% CI: −0.0472 to 0.0466), indicating potential publication bias (Figure 5). The trim-and-fill method (L0 estimator) identified 7 missing studies on the right side (SE = 3.5632). Adjusted analysis resulted in an SMD of −0.40 (95% CI: −0.54 to −0.26, *p* < 0.0001), with *I*^2^ = 89.11% and Q = 321.3465 (df = 35, *p* < 0.0001) (Figure 6), indicating that bias may have inflated the original estimate, although the effect remained significant.

### 3.7 Sensitivity analyses

**Figure 7:**
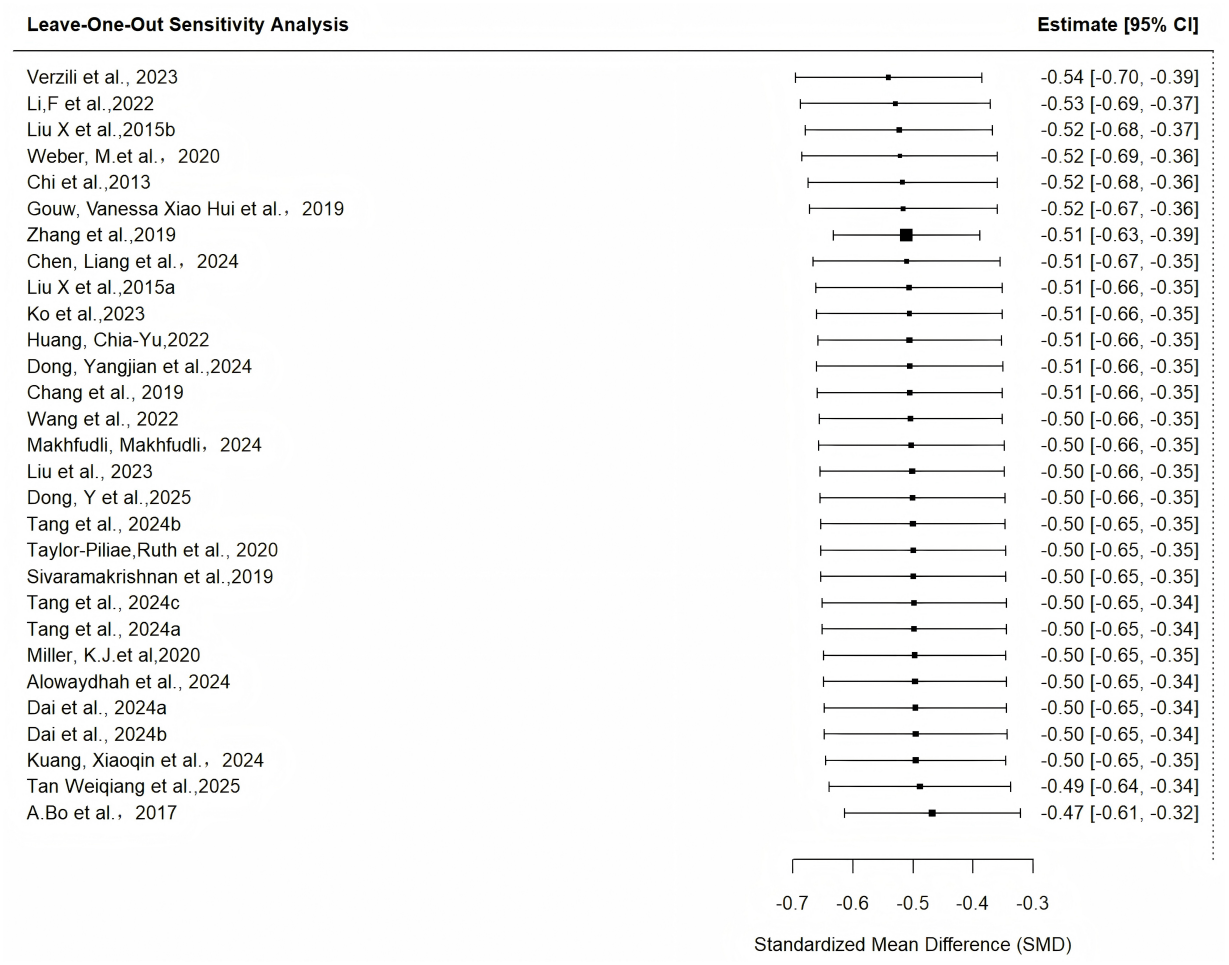
Leave-one-out sensitivity analysis.

The leave-one-out sensitivity analysis confirmed robustness, with SMDs ranging from −0.47 to −0.53, and 95% CIs consistently excluding zero (Figure 7). Exclusion of studies with SMD > |2| (none meeting criteria) resulted in an unchanged SMD of −0.51 (95% CI: −0.66 to −0.36, *p* < 0.0001), with *I*^2^ = 90.89%, confirming the stability of the findings.

**Figure 8:**
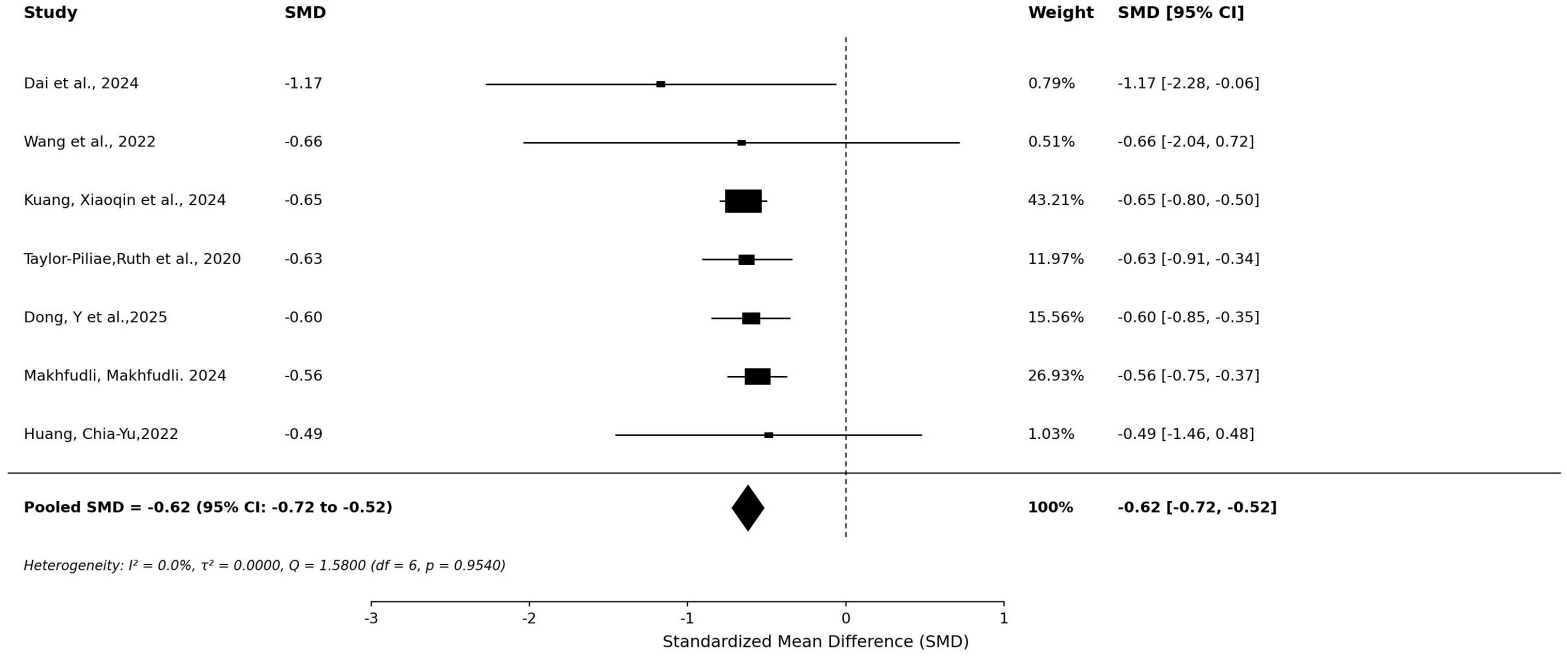
Forest plot of quality-stratified sensitivity analysis restricted to High Confidence reviews.

To evaluate the influence of methodological quality on pooled estimates, a sensitivity analysis was conducted restricting the sample to the 7 reviews rated as High Confidence by AMSTAR-2 (Dai et al., 2024; Wang et al., 2022; Dong et al., 2025; Makhfudli et al., 2024; Kuang et al., 2023; Huang et al., 2022; Taylor-Piliae and Finley, 2020). The pooled SMD was −0.62 (95% CI: −0.72 to −0.52, *p* < 0.0001; Figure 8), with no statistically significant heterogeneity detected (*I*^2^ = 0.0%, τ^2^ = 0.000, Q = 1.580, df = 6, p = 0.954). The effect was stronger and fully homogeneous compared to the full-sample analysis, suggesting that a substantial portion of the heterogeneity observed in the full analysis may be attributable to methodological variability among lower-quality reviews, rather than genuine clinical differences in MBE efficacy.

## 4 Discussion

This umbrella meta-analysis, synthesizing 25 systematic reviews and meta-analyses involving over 20,000 older adults, provides preliminary evidence, subject to substantial heterogeneity, that MBE may alleviate depressive symptoms with a pooled effect of moderate magnitude. Given the high *I*^2^, this estimate should be interpreted as reflecting a general directional trend across diverse study contexts rather than a precise effect size. Notably, the quality-stratified sensitivity analysis restricted to 7 High Confidence reviews yielded a stronger and fully homogeneous estimate (SMD = −0.62, *I*^2^ = 0.0%), suggesting that a substantial portion of the heterogeneity in the full analysis may reflect methodological variability among lower-quality reviews. This restricted estimate (SMD = −0.62) represents a clinically meaningful magnitude of improvement by Cohen's criteria. It is noteworthy that existing evidence for antidepressant efficacy in older adults with comorbidities raises tolerability concerns (De Luca et al., 2025), and a systematic review found limited high-quality evidence supporting antidepressant effects on standardized depression scales in older adults, including those with dementia or cognitive decline (Dudas et al., 2018). In this context, MBEs may represent a valuable complementary or alternative option—particularly given their favorable safety profile—though direct head-to-head comparisons with pharmacotherapy are lacking and equivalence cannot be inferred from the present data.

High heterogeneity persisted across Tai Chi and Yoga subgroups, likely due to differences in study populations (e.g., comorbidities like chronic heart failure or mild cognitive impairment) and methodological variations. Notably, the Qigong subgroup demonstrated not only the largest pooled effect size (SMD = −0.49) but also the lowest heterogeneity (*I*^2^ = 11.4%) among the three modalities, suggesting that the evidence for Qigong's antidepressant effect in older adults is both meaningful and internally consistent, though this finding should be interpreted with caution given the small number of included reviews (*n* = 5) in this subgroup. Publication bias was evident, with trim-and-fill adjustment yielding a slightly attenuated but still statistically significant effect, suggesting potential overestimation in unadjusted estimates. Sensitivity analyses, including leave-one-out and outlier exclusion, confirmed the robustness of these findings.

Notably, only one subgroup analysis by intervention type was conducted, as pooling across other dimensions (e.g., duration, intensity) was precluded by substantial variability within the included meta-analyses. These reviews included heterogeneous intervention protocols and comorbid conditions, making generalized subgrouping inappropriate and risking biased interpretations. This cautious approach prioritizes methodological integrity over exploratory analyses.

Overall, these results support the therapeutic role of MBE in geriatric depression, providing a non-pharmacological option with moderate evidence certainty, though tempered by heterogeneity and bias.

The pooled SMD from this umbrella meta-analysis indicates a moderate reduction in depressive symptoms among older adults engaging in MBE, aligning with prior syntheses on non-pharmacological interventions. This effect size is comparable to that reported in a meta-analysis of randomized controlled trials (RCTs) examining exercise for depression in older adults, which found a large antidepressant effect (Bigarella et al., 2022). However, that study included broader exercise modalities and noted similar heterogeneity. Our findings extend these by focusing specifically on MBE, revealing a consistent moderate benefit despite high *I*^2^, which may reflect variations in intervention delivery and population characteristics. The persistence of heterogeneity highlights the challenge of synthesizing diverse studies, a common issue in geriatric mental health research where comorbidities and functional limitations vary widely.

In comparison with previous studies, our results resonate with research on MBE specifically tailored for older adults. For instance, a systematic review and network meta-analysis demonstrated that MBE, including Tai Chi and Yoga, yield positive effects on both anxiety and depression (Li et al., 2020). This consistency highlights the efficacy of these practices in geriatric populations, particularly those with comorbidities, where physical limitations may favor low-intensity modalities over high-intensity alternatives. Another study further supports this, demonstrating the effectiveness of MBE in reducing depressive symptoms in older adults with chronic diseases, attributing benefits to improved self-efficacy and social engagement (Tang et al., 2024). Our analysis builds on this work by aggregating multiple meta-analyses, providing higher-level synthesized evidence and reinforcing MBE as viable options for symptom management in aging cohorts.

Compared to pharmacological treatments, MBE may offer a complementary option with a more favorable safety profile, though direct comparative evidence is lacking. A systematic review found that antidepressants have limited high-quality evidence for efficacy on depression rating scales in older adults with dementia (Dudas et al., 2018). Moreover, antidepressant treatments cause more adverse effects than placebos. A recent network meta-analysis suggests that, while antidepressants are effective for patients with comorbid medical conditions, their tolerability remains a concern (De Luca et al., 2025). Several studies suggest that MBE can be an effective adjunct or alternative intervention for mental health issues and other chronic diseases in older adults (Wang et al., 2022; Makhfudli et al., 2024; Tian et al., 2024).

Subgroup analyses identified Qigong as the most effective modality, surpassing Tai Chi and Yoga. A 2020 systematic review specifically on Qigong for depression in older adults reported that Qigong was the only treatment with a significant effect on depression severity, corroborating our subgroup findings and highlighting Qigong's advantage in culturally resonant contexts (Gill et al., 2020). For Yoga and Tai Chi, our results indicate that the intervention effect is significant, though less pronounced than that of Qigong. These subgroup insights affirm Qigong's potential as an optimal intervention, warranting prioritization in clinical recommendations for older adults. Research indicates that Qigong interventions can enhance older adults' self-control and self-efficacy via social learning (Levasseur et al., 2009). Qigong emphasizes restoring the flow of “Qi” via mind-body regulation, thereby enhancing physical and mental health (Zhang et al., 2016; Bandura, 1986). This psychophysiological self-regulation mechanism may help alleviate psychological symptoms such as depression (Gouw et al., 2019). Research by Liu et al. further sheds light on this underlying mechanism (Liu et al., 2023), showing that MBE such as Qigong, which combine low-intensity muscular activity with internally guided mindfulness, can reduce stress and inflammation, enhance brain network connectivity and neuroplasticity, and positively modulate oxidative stress, salivary cortisol, and gamma-aminobutyric acid (GABA) levels (Streeter et al., 2020; Yadav et al., 2019). Collectively, these physiological and neural improvements may enhance brain function, thereby improving mood and alleviating depressive symptoms.

Publication bias was evident in our analysis, as indicated by the asymmetric funnel plot and a significant Egger's test. The trim-and-fill method identified 7 missing studies on the right side, the adjusted pooled SMD suggests that the initial estimate may have been inflated by the selective reporting of positive results, a common issue in non-pharmacological intervention studies. Despite the adjustment, the effect remained statistically significant, though attenuated, suggesting that MBE continue to provide meaningful benefits, albeit more modestly than unadjusted estimates suggest.

This umbrella meta-analysis offers a high-level synthesis of 25 reviews, providing greater evidentiary weight than individual meta-analyses. Strengths of this analysis include a comprehensive search strategy across multiple databases, rigorous adherence to PRISMA and PRIOR guidelines, and the use of advanced bias correction (trim-and-fill) and sensitivity analyses, which confirmed the robustness of the findings.

Our findings are subject to several limitations. First, this umbrella meta-analysis employs second-order pooling of effect sizes across meta-analyses, a method that assumes a degree of independence among reviews not fully guaranteed by CCA alone, as overlapping primary studies may receive differential weighting across reviews. The pooled SMD should therefore be interpreted as a synthesis-level directional estimate, and conclusions should be considered in conjunction with the narrative synthesis. This approach follows precedent in the umbrella meta-analysis literature (Bigarella et al., 2022; Singh et al., 2025) and the moderate CCA (6.98%) supports its application here. Second, substantial heterogeneity persisted across analyses, likely attributable to variations in intervention protocols, durations, and participant comorbidities, as reflected in baseline characteristics. This precludes definitive conclusions on optimal modalities. Third, publication bias was evident, with trim-and-fill adjustment reducing the effect size, suggesting a potential overestimation in the included reviews, particularly those with low AMSTAR-2 ratings. Fourth, reliance on review-level data limits granularity, as primary study quality was not reassessed. Additionally, 18 of 25 reviews originated from Asia, potentially limiting generalizability to diverse populations. GRADE ratings were moderate overall but low for several reviews due to methodological limitations (e.g., nine critically low ratings in AMSTAR-2). Fifth, the included MBE interventions comprised only Tai Chi, Qigong, Yoga. Due to strict screening, additional MBE interventions could not be included. Finally, the absence of long-term follow-up data in most reviews hinders assessment of sustained effects. Future research should prioritize high-quality, large-scale randomized controlled trials with standardized intervention protocols to mitigate heterogeneity and bias. Incorporating diverse populations from non-Asian regions could enhance generalizability. Long-term follow-up assessments are essential to evaluate sustained effects, and exploring dose-response relationships (e.g., duration and intensity) may help identify optimal intervention regimens. Finally, preregistration of protocols and inclusion of unpublished data may help mitigate publication bias, thereby strengthening the evidence base for MBE in the management of geriatric depression.

This umbrella meta-analysis provides preliminary moderate-certainty evidence that MBEs, particularly Qigong, may be associated with meaningful reductions in depressive symptoms in older adults. These findings may carry practical implications for clinicians seeking non-pharmacological options to complement existing care, particularly given the tolerability concerns and limited high-quality evidence for antidepressants in older adults with comorbidities. MBEs are low-cost, accessible, and associated with minimal adverse effects, making them feasible candidates for integration into multidisciplinary care plans as adjunctive interventions. Among the modalities examined, Qigong demonstrated the most consistent evidence, followed by Yoga and Tai Chi, though clinicians should note that subgroup estimates are based on limited numbers of reviews and should not be interpreted as definitive rankings. Importantly, the present evidence base is characterized by high methodological heterogeneity, predominance of lower-quality reviews, and potential publication bias; clinical recommendations should therefore be made with appropriate caution and tailored to individual patient characteristics, preferences, and functional capacity. Standardized MBE protocols adapted to the physical and cognitive capacities of older adults, as well as community-based implementation models, represent important areas for future development. Further high-quality research establishing long-term efficacy and optimal dosing will be essential before stronger clinical guidance can be issued.

## 5 Conclusion

In conclusion, this umbrella meta-analysis synthesizing 25 systematic reviews provides moderate-certainty evidence that MBE are associated with a moderate reduction in depressive symptoms among older adults, though the substantial heterogeneity across included reviews (*I*^2^ = 90.89%) necessitates cautious interpretation of the pooled estimates, which should be regarded as reflecting a general directional trend rather than definitive effect sizes. A quality-stratified sensitivity analysis restricted to High Confidence reviews yielded a stronger and homogeneous effect (SMD = −0.62, *I*^2^ = 0.0%), suggesting that Qigong, in particular, may represent a promising non-pharmacological option for geriatric depression. Nevertheless, high heterogeneity, evidence of publication bias, and the predominance of lower-quality reviews temper the strength of these conclusions. Rigorous future RCTs with standardized protocols are needed to confirm these benefits and establish optimal intervention parameters. Pending such evidence, integrating MBE as a complementary component of depression management in aging populations warrants further investigation.

## Data Availability

The original contributions presented in the study are included in the article/supplementary material, further inquiries can be directed to the corresponding author.
